# Assessing the impact of sequencing platforms and analytical pipelines on whole-exome sequencing

**DOI:** 10.3389/fgene.2024.1334075

**Published:** 2024-05-16

**Authors:** Yanping Sun, Xiaochao Zhao, Xue Fan, Miao Wang, Chaoyang Li, Yongfeng Liu, Ping Wu, Qin Yan, Lei Sun

**Affiliations:** ^1^ GeneMind Biosciences Company Limited, Shenzhen, China; ^2^ Clinical Research Institute, Shanghai General Hospital, Shanghai Jiao Tong University School of Medicine, Shanghai, China

**Keywords:** WES, NovaSeq 6000, GenoLab M, HD832, SNP, InDel

## 1 Introduction

Next-generation sequencing (NGS) is transforming clinical applications from prenatal testing to tumor profiling and early cancer diagnosis, greatly improving our ability to identify novel disease-causing genetic mutations and conduct comprehensive diagnostic testing in the clinic (Reid et al., 2014; Merino et al., 2020). Whole-exome sequencing (WES) is a commonly used method that captures most coding regions from the genome for sequencing since these regions contain the majority of disease-causing mutations (Consortium, 2005). In 2011, the US Food and Drug Administration awarded Helix as a WES platform for a direct-to-consumer genetic risk test (Allyse et al., 2018). WES is becoming a rapid, cost-effective molecular diagnostic tool for patients with genetic diseases (Gahl et al., 2012; Gilissen et al., 2012; Linderman et al., 2014; Ombrello, Sikora, and Kastner, 2014). As the demand for WES increases and the cost of NGS decreases, WES, as a technique, requires a generous understanding of how experimental design can improve data interpretation and thus improve biological outcomes (Cherukuri et al., 2015).

In recent years, sequencers and corresponding *in silico* software have also been continuously updated (Hu et al., 2021). From the analysis perspective, processing steps mainly include quality assessment, read alignment, variable identification, and annotation. Various combinations of tools in each of the above sections lead to different performances among pipelines, ultimately affecting the interpretation of variation calling (Barbitoff et al., 2022). Therefore, in this study, we selected seven popular analysis pipelines to evaluate the influence of these pipelines on WES results.

NGS technology has significantly impacted the field of genomics since its first release in 2005 (Ben Khedher et al., 2022). Since then, many different NGS platforms have been developed (Pervez et al., 2022). Among them, NGS machines based on the Illumina sequencing by synthesis (SBS) method have dominated the sequencing market. NovaSeq 6000, the latest instrument, now generates 6 TB of sequence data per run, costing 10 USD/GB (Juhasz, 2021). The NextSeq 550 platform is also a prevailing WES sequencing platform (Hu et al., 2021). Although its cost is higher than that of the NovaSeq 6000, it affects its market share to a certain extent.

Recently, GeneMind Biosciences Company Limited launched a new sequencing instrument (GenoLab M ™) based on their previous work on the GenoCare™ single-molecule sequencer (Li et al., 2022), a promising sequencing platform with high performance and low cost. The GenoLab M sequencer employs SBS and reversible termination approaches, which promise to deliver high-quality sequencing data faster and at lower prices than Illumina’s sequencing instruments. The GenoLab M now generates 300 GB of sequence data in a single run, costing 6 USD/GB (Liu et al., 2021). The previous study has shown that GenoLab M can achieve results comparable to the Illumina platform in the transcriptome, but there are few relevant studies on WES (Li et al., 2022). In this study, we selected two market-proven platforms with high market share and one newly launched sequencing platform to study the impact of different sequencing platforms on WES results.

Horizon Discovery Group plc has announced that it has added a golden standard genotype dataset (sample HD832) published by the Horizon consortium that can be used to test the WES variants. Nevertheless, few studies on sample HD832 have been published so far. The reference standard HD832 contains over 380 variants across 152 essential cancer genes, and every batch of formalin-fixed paraffin-embedded (FFPE) has 25 variants confirmed by ddPCR. Therefore, we focused on the comparison results of SNPs and InDels of high-confidence variants, 24 variants confirmed by ddPCR, and then expanded all variants in this study. Unfortunately, there were no benchmarking variation sets as the gold standard sets of HD832. Therefore, this study selected HD832 as the test sample, providing a meaningful dataset.

WES has seen a marked increase in the application for detection of genetic diseases in recent years (J.-Y. Nam et al., 2016). For instance, in cases of intellectual disability, NGS technologies have markedly improved the identification of both established and novel genes implicated in such conditions. When combined with CNV analysis, WES can pinpoint a pathogenic variant in approximately 30% of patients (J. Wang et al., 2017). The current diagnostic success rates support the use of WES as a primary diagnostic tool, allowing for early diagnosis in intellectual disability or autism spectrum disorder patients and ultimately improving the quality of life for affected families (L.P. Bruno et al., 2021). The application of WES has also significantly bolstered the diagnostic accuracy for fetuses presenting with ultrasound abnormalities (Bianchi, 2019). In light of these advancements, we have included the standard HG001 sample in our WES analysis for genetic disease research.

In this study, our goal was to investigate the influence of the sequencing platform and pipeline on WES results and discover essential factors affecting WES by comparing the consistency of biological and technical replicates across different datasets.

## 2 Materials and methods

### 2.1 Library preparation and sequencing

Total DNA was extracted from the FFPE scroll of HD832 using the SEQPLUS FFPE DNA Isolation kit according to the manufacturer’s instructions. The FFPE product of HD832 was purchased from Mingma Technologies Co., Ltd., China. Then, 200 ng of genomic DNA was sheared using the Covaris LE220 ultrasonicator (Covaris) to the target of 150–200 bp average size. DNA libraries were prepared using the SureSelectXT Low Input reagent kit and SureSelectXT HS2 DNA reagent kit (Agilent). After the purification of beads, the pre-capture libraries containing exome sequences were captured using the SureSelect Human All Exon V8 kit (Agilent). Finally, the same library was split and loaded into NovaSeq 6000, GenoLab M, and NextSeq 550 using the PE150 sequencing mode. We used two HD832 standard samples (R1 and R2) to be sequenced on three sequencing platforms, respectively, and the standard sample R2 was repeated for sequencing three times on the GenoLab M and NovaSeq 6000 platforms. For the normal standard HG001, we used SureSelect Human All Exon V6 kit to construct one library. Then, the library was split and loaded into five platforms NextSeq 550, FASTASeq 300, NovaSeq 6000, GenoLab M, and SURFSeq 5000.

### 2.2 Read alignment and variant calling

The raw reads of nine data were filtered and trimmed by fastp using default parameters. The filtered reads were aligned to hg19 via “Sentieon BWA” of Sentieon software v202112.04, and sorting was performed using the “sort” utility tool. Then, “LocusCollector” and “DeDup” tools were used to remove duplicate reads, and the de-duplicated BAM files were used for calling variants. Quality metrics were generated from these BAM files using the FastQC and bamdst tools. We screened out several of the most used pipelines for variant calling (Table 1). Variants were identified by seven variant calling pipelines: SAMtools–VarScan2 (v1.10 and v2.3.9) (Koboldt et al., 2009; Koboldt, Larson, and Wilson, 2013), SNVer (v0.5.3) (Wei et al., 2011), Sentieon-TNscope (Freed, Pan, and Aldana, 2018), GATK-HaplotypeCaller (v4.2.6.1) (Ren, Bertels, and Al-Ars, 2018), bcftools-mpileup (v1.14) (Lefouili and Nam, 2022), Strelka2 (v2.9.7), (Kim et al., 2018), and GATK-Mutect2 (v4.2.6.1) (Kiniwa et al., 2019). For the VarScan2, TNscope, and HaplotypeCaller pipeline, the variants were filtered using FPfilter, FilterMutectCalls, and VQSR tools, respectively. For all pipelines, the parameters DP ≥ 20 and AD ≥ 3 were applied. For the GATK-HaplotypeCaller algorithm, following the best practice guidelines, raw variants were filtered by GATK VariantRecalibrator using the 1,000 G, HapMap, Omni, dbSNP, and Mills InDels datasets. We focused on the usage of the tumor-only model for the Mutect2 pipeline. For other five pipelines, we used default parameters to call variants, excepted -b 0.1 for SNVer. For HG001, we aligned the sequencing reads using BWA-MEM to the hg19 reference genome, and Picard (v2.27.5, https://broadinstitute.github.io/picard/) was used to mark duplicates in the BAM files. Quality metrics were generated from these BAM files by the fastQC and bamdst tools.

**TABLE 1:** Lists of variant callers used in this study.

ID	Tool	Version	Main property	Literature citation	Link	Sample
1	VarScan2	v2.3.9	Applying a heuristic algorithm determines the genotype for normal and tumor samples independently based on adjustable minimum thresholds for coverage, base quality, variant allele frequency, and statistical significance. VarScan 2 is used for the detection of somatic mutations and copy number alterations in exome data	4,533	https://varscan.sourceforge.net/	HD832–HG001
2	SNVer	v0.5.3	Calling common and rare variants in analyzing pooled or individual NGS data. It formulates variant calling as a hypothesis testing problem and uses a binomial–binomial model to test the significance of observed allele frequency against the sequencing error	317	https://snver.sourceforge.net/	HD832–HG001
3	Sentieon-TNscope	v202112.04	In the ICGC-DREAM Somatic Mutation calling challenge, TNscope is the leader in accuracy for SNPs and InDels, by combining the improvements in the variant caller with machine learning	40	https://support.sentieon.com/appnotes/tnscope_ml/	HD832
4	GATK-Mutect2	v4.2.6.1	Mutect2 is a somatic variant caller that uses local assembly and realignment to detect SNPs and InDels. Mutect2 supports the normal-tumor mode and tumor-only mode	323	https://github.com/broadinstitute/gatk/releases	HD832
5	GATK-HaplotypeCaller	v4.2.6.1	A popular set of programs for discovering and genotyping variants from NGS data	22,657	https://github.com/broadinstitute/gatk/releases	HD832–HG001
6	bcftools-mpileup	v1.14	SAMtools implements various utilities for post-processing alignments in the SAM format, such as indexing, variant caller, and alignment viewer. In particular, SAMtools mpileup (now Bcftools mpileup) was previously the most widely used variant caller	48,024	https://samtools.github.io/bcftools/howtos/index.html	HD832–HG001
7	Strelka2	v2.9.7	Strelka2 introduces a novel mixture-model-based estimation of insertion/deletion error parameters from each sample, an efficient tiered haplotype-modeling strategy. Strelka2 software is claimed to be time-efficient, which is a very important aspect of clinical usage	872	https://github.com/Illumina/strelka	HD832–HG001

### 2.3 Comparison of results with truth sets and ddPCR sets

The truth sets and the ddPCR sets of HD832 were downloaded from https://horizondiscovery.com/en/reference-standards/products/oncospan-gdna. There are 234 SNPs and 23 InDels in Agilent V8 regions, and 24 variants are in the ddPCRs sets. The variants’ AF ranges from 1% to 100%. This dataset was designed as a truth set for following benchmarking analyses to evaluate all variation sets. Precision, recall (sensitivity), and F-score were calculated. The following formulas were used.

$$\text{Precision}:\text{TP}/\left(\text{TP}+\text{FP}\right),$$

$$\text{Sensitivity}:\text{TP}/\left(\text{TP}+\text{FN}\right).$$

F-score: 2*Sensitivity*Precision/(Sensitivity + Precision).

TP, true positive; FN, false negative; FP, false positive.

For HG001, five variants’ callers, which included SNVer, Strelka2, VarScan2 (filtered by FPfilter), GATK-HaplotypeCaller (filtered by VQSR), and bcftools-mpileup, were used to detect SNVs and InDels with default parameters. All VCF files were compared against the GIAB benchmark dataset (v4.2.1) using hap.py v0.3.15 for accuracy evaluation.

### 2.4 Performance comparison of all SNP and InDel sets for HD832

To reduce false-positive calls, we filtered the variants. For the VarScan2, TNscope and HaplotypeCaller pipelines, the variants were filtered by FPfilter (with--min-var-count 3), FilterMutectCalls, and VQSR (best practice guideline parameters) tools, respectively. For all pipelines, the parameters read depth (DP) ≥ 20, allele depth (AD) ≥ 3, and allele frequency ≥0.05 were applied. The mutation results from the VarScan2 and SNVer pipelines were compared. All the samples, including sample R1 (GL_R1 and NV_R1) and sample R2 (NV_R2.1, NV_R2.2, and NV_R2.3), were biologically replicated samples. Then, the performances of variants were assessed across three platforms (GL: GenoLab M, NS: NovaSeq 6000, and NX: NextSeq 550), where the variants of GL and NS platforms, respectively, took the intersection of repeated samples as their own variant sets. Furthermore, the variants were annotated for impact prediction using ANNOVAR software. We filtered the common mutations which were recorded by the four-frequency databases (Genome Aggregation Database (gnomAD), 1000 Genomes Project (1000G), Exome Aggregation Consortium (ExAC), and Exome Sequencing Project v6500 (ESP6500)) with the minor allele frequency (MAF) greater than 0.1%.

## 3 Results

### 3.1 Data quality check

We obtained nine WES datasets by sequencing gDNA from HD832. The average rates of quality scores above Q30 in GenoLab M, NovaSeq 6000, and NextSeq 550 were 91.48%, 92.26%, and 93.15%, respectively. GenoLab M had a minor performance gap compared to NovaSeq 6000 and NextSeq 550 (Supplementary Table S1). The mapping rates for all samples were above 98%. However, the duplication rate in GenoLab M was less than one-third of that in NovaSeq (8.18% VS 21.54%). Furthermore, the target coverage over 30X in NovaSeq 6000 and NextSeq 550 was higher than that in GenoLab M, while the coverage depth was relatively similar in the three sequencing platforms.

### 3.2 Performance comparison of high-confidence variants in HD832

**FIGURE 1:**
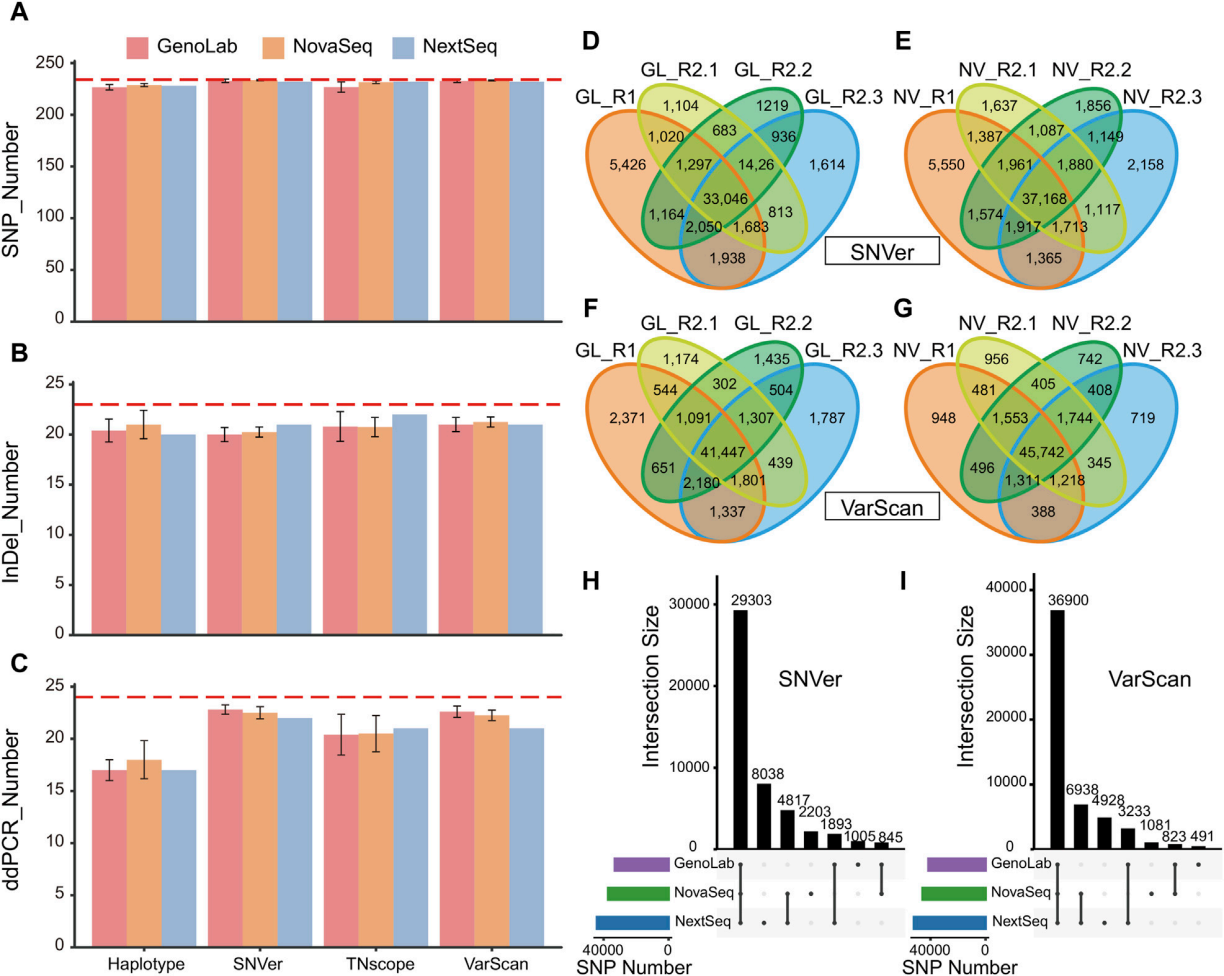
Comparative analysis of the performance of high-confidence variants for three platforms: **(A)** comparison of high-confidence variants in SNP; **(B)** comparison of high confidence variants in SNP; **(C)** comparison of variants confirmed by ddPCR in all datasets (red dashed lines represent the number of truth sets); **(D)** SNP sets of SNVer in the GenoLab M platform; **(E)** SNP sets of VarScan2 in the GenoLab M platform; **(F)** SNP sets of SNVer in the NovaSeq 6000 platform; **(G)** SNP sets of VarScan2 in the NovaSeq 6000 platform; **(H)** upset diagram of SNP calling results by SNVer pipeline analysis of nine datasets; and **(I)** upset diagram of SNP calling results by VarScan2 pipeline analysis of nine datasets. Note: GATK-HC, GATK-HaplotypeCaller; NS, NovaSeq 6000; GL, GenoLab M; NX, NextSeq 550.

First, the detection numbers of high-confidence variants on three platforms via seven pipelines were obtained. Four tools with the best results were selected for display because they were able to detect more high-confidence variants. It was found that, under the same software, the difference in the number of detected variants among different platforms was very negligible (Figures 1A, B). Under the pipeline VarScan2, the average SNP detection numbers on the three platforms were 233, 232, and 234, respectively. Although all platforms were not able to detect all high-confidence variants, we calculated the F-score and Recall of all samples under different analysis tools (Supplementary Table S2). At the same time, the results show that there are large differences in high-confidence variants among tools. For example, the same dataset detected 193 SNPs in Strelka2, but 234 SNPs were detected in VarScan2. It is similar in the set of variants confirmed by ddPCR (Figure Figure1C). Since the number of InDels given by the high-confidence variants was small, the difference between platforms was 1–2 InDels (Figure 1B), but the difference among tools was up to 7 InDels. In addition, SNVer and VarScan2 performed best in the detection rate of high-confidence variants. We further collected a set of novel variants through the intersection of our 63 datasets (Supplementary Table S3), which potentially cause significant impacts, beyond the truth sets published by HD832. Given that these novel variants are consistently detected across various sequencing platforms and software, they present themselves as promising candidate variants. Once they were verified by Sanger sequencing, it is advisable to incorporate them into the true set. This integration would enrich the dataset with verified loci, thereby enhancing the accuracy and reliability of future genetic analyses.

### 3.3 Performance comparison of all SNPs and InDels in HD832

In comparing the results of the high-confidence variant set, the platform has a minor influence on the WES results. The small number of reference sets might have an impact, so all the variants were calculated. All nine datasets were used for calling variants via the two outstanding SNVer and VarScan2 pipelines to explore the effects of biological and technical replicates on WES results. The number of overlapping mutations in the nine datasets was 29,303 in SNVer and 36,900 in VarScan2 (Supplementary Figures S1A, S1B). The number of mutations detected by two software applications was quite different, and the proportion of overlap in each sample was also quite different. For example, the sample proportion of NV-R1 was 55.67% in SNVer, and the proportion in VarScan2 was 70.78% (Supplementary Figure S1). It was consistent with the results of the high-confidence variant set. Moreover, the sample with the smallest overlap mutation was NV-R1, with only 55.67%, while the highest proportion was 71.35% (SNVer). For InDel results, the situation was even worse, and the sample with the highest proportion of overlap was only 55.77% (Supplementary Figure S2). These results indicated that the agreement among the nine datasets was poor in SNPs and InDels.

### 3.4 Comparison of technical and biological replicates in HD832

To further explore the reasons for the differences among the nine samples, we separately counted the overlap variants of the sequencing platform. Eight datasets from the GenoLab M and NovaSeq 6000 platforms were chosen for subsequent analysis. In this, R1 and R2 were biological replicates derived from two standard HD832 libraries, while R2.1, R2.2, and R2.3 were technical replicates derived from three sequencing results of the same library. For the SNVer pipeline, the proportion of shared SNPs between the biological replicates was only 69.39% in GenoLab and 70.61% in NovaSeq, while the proportion of technical replicates was only 75%–81% (Figures 1D–G). For InDels, the proportion of shared mutations between biological replicates was 61.77% and 65.2%. In comparison, the proportion of shared mutations between technical replicates ranged from 65% to 70.81% (Supplementary Figure S3). The consistency of technical replicates was higher than that of biological replicates. The results of VarScan2 were slightly better than those of SNVer, especially in the results of the NovaSeq platform, in which the ratio of the overlapping sites of SNPs exceeds 87% between biological replicates and technical replicates, respectively. The proportion of overlapping sites in InDels also exceeded 71%. However, the ratio of overlapping sites in InDels on the GenoLab M sequencing platform decreased to 57%–61%. On the contrary, the proportion of SNPs increased to 80.6%–86.16% (Figures 1D–G). The results showed that there were still differences between the sequencing results of the biological replicates and the technical replicates from the standard sample HD832.

### 3.5 Comparing differences across sequencing platforms in HD832

To further study the impact of sequencing platforms on WES results, the intersection of the data on four samples from the same sequencer was taken. It showed that the results after taking the intersection were improved compared with the results of the nine samples. The concordance of SNPs on the GenoLab and NovaSeq platforms reached 88.67% and 78.84% in SNVer and 89.03% and 80.67% in VarScan2, respectively (Figures 1H, I). The consistency of InDels reached 69.76% and 53.93% in VarScan2, and 78.73% and 71.05% in SNVer, respectively. In the NextSeq platform, owing to the lack of biological replicates, SNPs accounted for only 66.52% in SNVer and 70.96% VarScan2, respectively (Supplementary Figure S4). These results suggest that biological replicates could improve the result accuracy.

### 3.6 Comparing differences across sequencing platforms in HG001

To further validate our findings in genetic disease, we conducted a study to investigate the impact of analysis software on WES results in comparison to sequencing platforms. Our results indicated minimal variation in F-values across platforms (Supplementary Figure S5). Two newly launched sequencing platforms, namely, FASTSeq 300 and SURFSeq 5000, were incorporated, and two tools that were more suitable for tumor detection were phased out (Supplementary Figure S6). The results underscore that under the identical analysis pipeline, the disparity in SNP detection among various sequencing platforms is minimal. Specifically, for mpileup, the F-score across platform ranged from 0.8738 to 0.8856 (Supplementary Table S4). Nevertheless, a more pronounced divergence was observed in the detection of InDels. Illustratively, the underperforming SNVer pipeline reported an average F-score of a mere 40%, in stark contrast to the superior Strelka2 pipeline, which boasted an average F-score of 76%. These insights suggest that the choice of the sequencing platform exerts a negligible influence on the WES outcomes for the HG0001 sample. Conversely, the analysis software application, while having a muted effect on SNP detection, significantly impacts InDel identification. In contrast to the HD832 tumor detection, SNVer performed worst and Strelka2 was best in HG001.

## 4 Discussion

This study used the standard sample HD832 for WES to produce multiple datasets by three sequencing platforms, seven analysis pipelines, and HG001 for genetic disease to produce multiple datasets by five sequencing platforms and five analysis pipelines. By comparing these datasets, it was found that the analysis pipeline had a greater influence on the WES results than the sequencing platform. At the same time, there were still differences between the technical replicates of the same sequencing platform. Biological replicates have worse concordance results than technical replicates. This study provides the following suggestions for improving the accuracy of WES results: first, it is essential to select an appropriate analysis pipeline for WES projects; second, the sequencing platforms have little impact on WES results; in addition, biological replicates are necessary for WES research and should be considered in addition to adding technical replicates of critical samples. Unfortunately, this study failed to use more sequencing platforms for comparison, such as BGI, PacBio, and Nanopore (Jeon et al., 2021). Since HD832 only has small benchmarking variant sets, we could not compare the differences between the three sequencing platforms using statistical methods such as F1-score. It is recommended that future studies consider using the inheritance state consistency analysis (ISCA) method to enhance the reliability of results (J.C. Roach et al., 2010). Nevertheless, this study aims to initially explore the potential factors affecting the results of WES. In the subsequent research, we will collect more samples and use sequencing platforms to explore these impact factors in depth.

As costs continue to decline, more and more researchers and clinicians are using NGS to analyze clinical samples (Schwarze et al., 2018). Currently, several sample processing protocols, library preparation methods, sequencing technologies, and bioinformatics pipelines have been used to identify cancer-associated mutations (Xiao et al., 2021). In addition, samples arriving at the testing laboratory may be in a different state (FFPE or fresh samples), and the amount of DNA input may be variable in clinical settings. Tumor purity rarely varies consistently across samples, posing enormous challenges for sequence analysis, instrumentation, and analytical tools (Xiao et al., 2021). Previous studies have found differences in the detection of SNPs and InDels between different pipelines (Hwang et al., 2015; Torkamaneh, Laroche, and Belzile, 2016; Xiao et al., 2021). Therefore, we used seven pipelines for variant calling. However, the result via the Genome Analysis Toolkit (GATK) has not yet exhibited in this study, owing to its worse result than VarScan2, SNVer, or Sentieon. Nevertheless, optimizing analytical pipelines and polishing sequencing platforms can improve WES results (Xiao et al., 2021). Therefore, developing new sequencing platforms and analyzing pipelines would benefit WES research.

Previous studies have shown differences between WES biological replicates of the same sample (Shi et al., 2018; Xiao et al., 2021). A few studies on the technical replicates of sequencing platforms were performed (Chen et al., 2019; Kim et al., 2021). However, we found differences between technical replicates, suggesting that the stability of current sequencing platforms still needs improvement. Encouragingly, GenoLab M, a newly released sequencing platform, had a small gap in terms of sequencing stability compared to NovaSeq. For NovaSeq, after several years of upgrading (Modi et al., 2021), the consistency of the platform can reach 87% under a specific analysis pipeline. Various factors can affect the accuracy of WES data, such as the target enrichment kits used, target sequence GC bias, PCR amplification bias, duplications and spurious genes, and other experimental design variables (Cherukuri et al., 2015; Tian et al., 2016). These factors directly and systematically affect the sensitivity of WES. Although developmental versions of exome enrichment kits continue to address these biases, the impact of experimental design on engineered replication in lineage-based WES still needs to be fully understood. Therefore, technical replication was emphasized while we focused on the sequencing platform. The results showed a great relationship between the technical duplication of the platform and the analytical pipeline, so the technical duplication of essential samples should be enrolled while increasing the analytical pipeline. For example, the number of technical replicates was expanded for reference negatives and critical family samples. Finally, we consider our method and results consistent with other studies that have noted benefits with replicate comparisons for variant detection and variant calling accuracy (Zhang et al., 2014; Cherukuri et al., 2015).

## 5 Conclusion

In summary, through comparative analysis of multiple datasets, we found that the analysis pipeline had a greater influence on the results of WES for HD832 and HG001 than the sequencing platform. SNVer and VarScan2 can obtain the best analysis results among the seven pipelines in HD832. Strelka2 can obtain the best analysis results among the five pipelines in InDel calling of HG001. In addition, we provided multiple practical reference datasets for the new sequencing platform and the standard HD832.

## Data Availability

The datasets presented in this study can be found in online repositories. Accession numbers are CNP0003701 (https://db.cngb.org/search/project/CNP0003701/) and CNP0005581 (https://db.cngb.org/search/project/CNP0005581/) in CNGBdb database.
