# Supplementary material for: Assessing the impact of sequencing platforms and analytical pipelines on whole-exome sequencing
Source: Front Genet. 2024 May 16;15:1334075. doi: 10.3389/fgene.2024.1334075 (PMC11137314; doi:10.3389/fgene.2024.1334075)
Supplement: Supplementary file 7 [file DataSheet1.DOCX]

Supplementary Material

1.Supplementary Figures and Tables


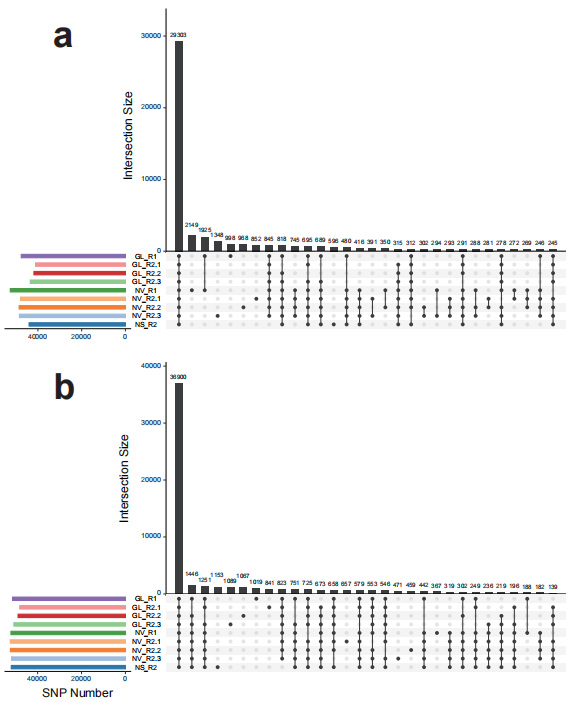


Figure S1. Upset diagram of variant calling SNP results of combinations in nine datasets. a. results using SNVer pipeline; b. results using VarScan2 pipeline. NS, NovaSeq 6000; GL, GenoLab M; NX, NextSeq 550; Groups R1 and R2 are biological replicates. R2.1, R2.2 and R2.3 are technical duplications.


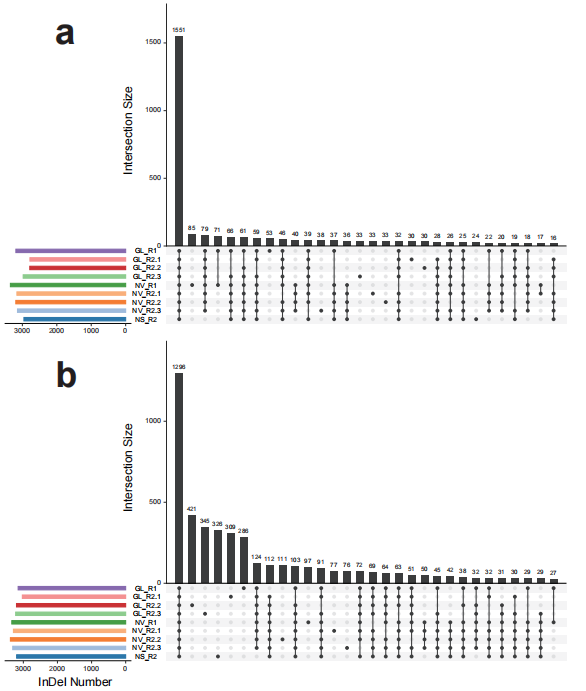


Figure S2. Upset diagram of variant calling InDel results of combinations in 9 datasets. a. results using SNVer pipeline analysis; b. results using VarScan2 pipeline analysis. NS, NovaSeq 6000; GL, GenoLab M; NX, NextSeq 550; Groups R1 and R2 are biological replicates. R2.1,R2.2 and R2.3 are technical duplications.


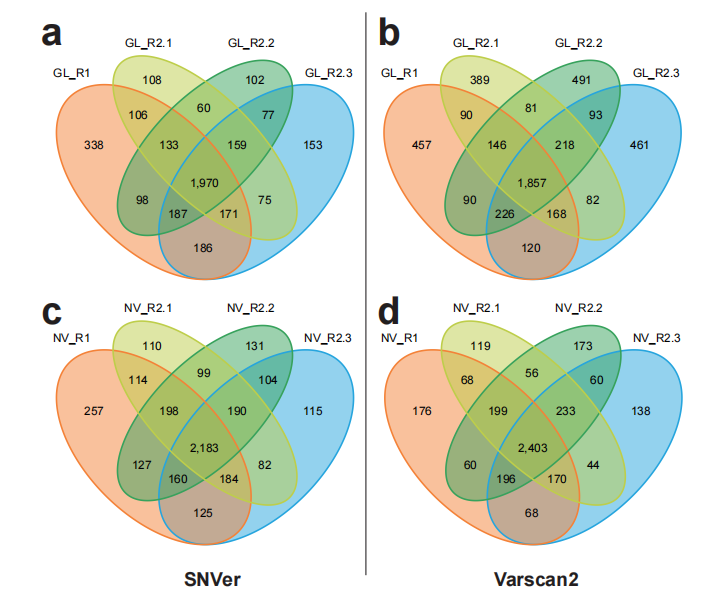


Figure S3. Venn diagram of variants calling performances in GenoLab M and NovaSeq 6000 platforms. a. InDel sets of SNVer in GenoLab M platform; b. InDel sets of VarScan2 in GenoLab M platform; c. InDel sets of SNVer in NovaSeq 6000 platform; d. InDel sets of VarScan2 in NovaSeq 6000 platform. NS, NovaSeq 6000; GL, GenoLab M; Groups R1 and R2 are biological replicates. R2.1, R2.2 and R2.3 are technical duplications.


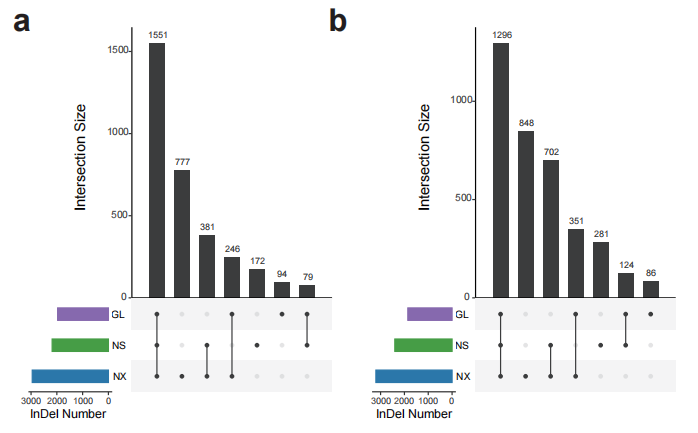


FigureS4. Upset diagram of variant calling InDel results of combinations in three platforms. a. results using SNVer pipeline; b. results using VarScan2 pipeline. NS, NovaSeq 6000; GL, GenoLab M; NX, NextSeq 550.


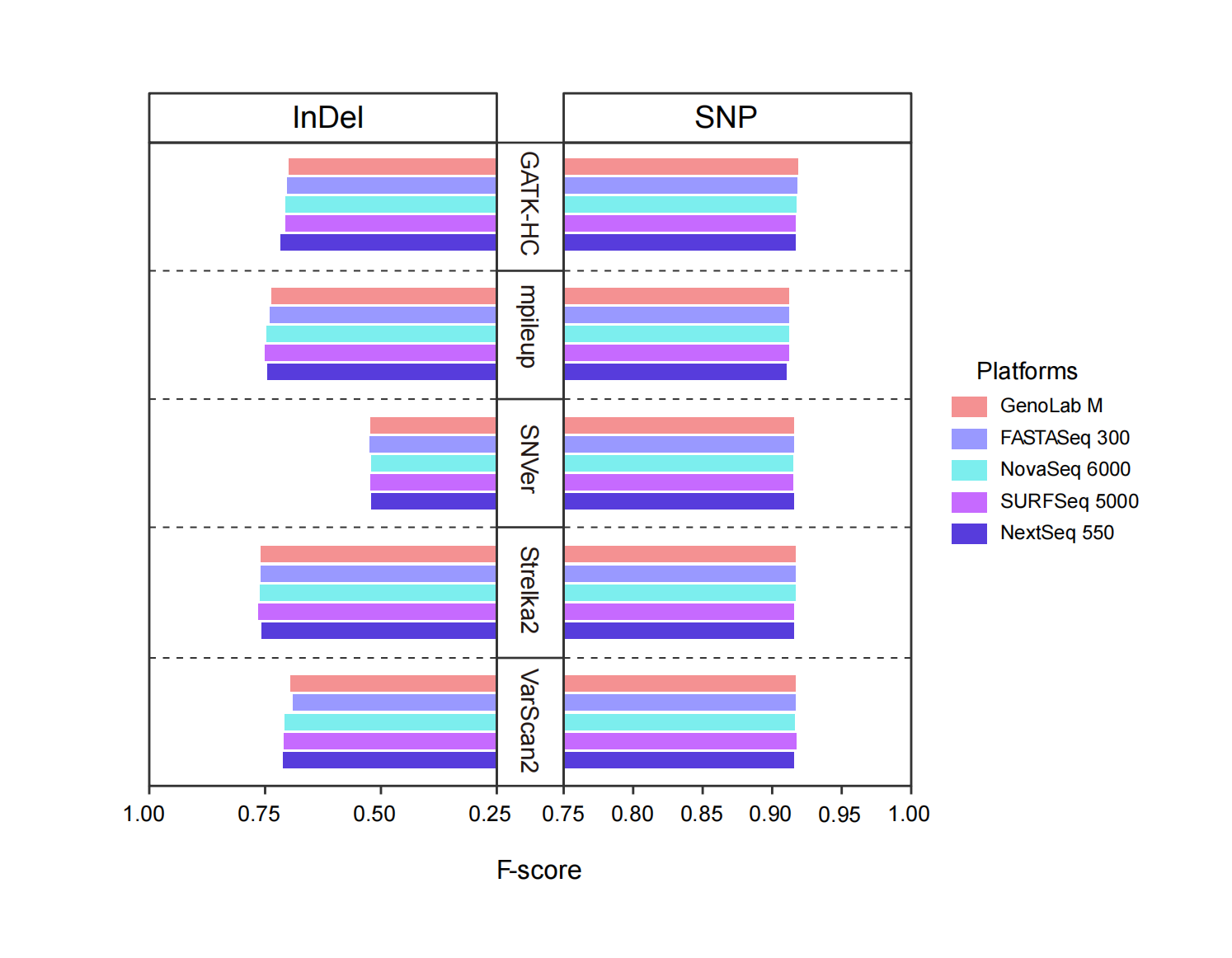


Figure S5. Comparison of F-score for SNPs (Right) and InDels (Left) detected across five callers and five platforms. GATK-HC means GATK-HaplotypeCaller, and mpileup means bcftools-mpileup.


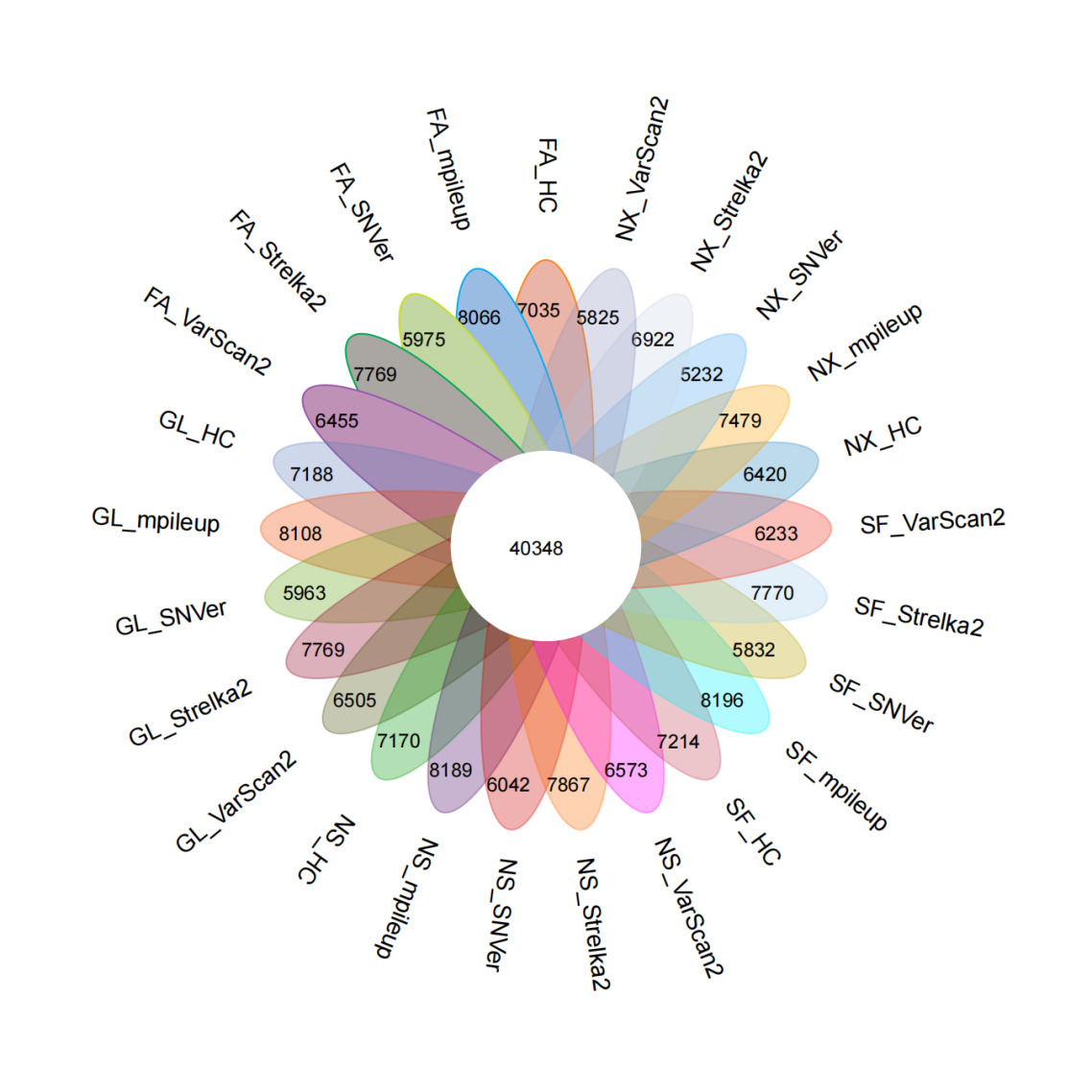


Figure S6 Venn diagram of truth variants calling performances in all sequencing platforms and all pipelines. NS, NovaSeq 6000; GL, GenoLab M; FA, FASTASeq 300; SF, SURFSeq 5000; NX, NextSeq 550. HC means GATK-HaplotypeCaller and mpileup means bcftools-mpileup.

The following tables are large datasets, so we have uploaded them as additional files. https://doi.org/10.5281/zenodo.11090742

Table S1. Summary of sequencing data for all datasets in this study. (Note: NS, NovaSeq 6000; NX, NextSeq 550; GL, GenoLab M; FA, FASTASeq 300; SF, SURFSeq.)

Table S2 The variants calling performances among sequencers and tools for sample HD832. (Note: NS, NovaSeq 6000; NX, NextSeq 550; GL, GenoLab M. Groups R1 and R2 are biological replicates. R2.1, R2.2 and R2.3 are technical duplications. Known_number, The number of variants considered to be of high confidence in the captured regions. The F-score of all datasets were calculated based on the list of high confidence carants published by OncoSpan Standard. )

Table S3. Novel variant genes identified in our study. (Note: The variants are beyond the list of the high confidence variants published by OncoSpan FFPE HD832. Chr, Chromosome; POS, Position; Ref, Reference; Alt, Alteration; Func, Functional consequences of the variant by ANNOVAR; Average Depth(×), The average depth for all datasets; Average AF, The average Allele frequency for all datasets. Gene names are listed in italics.)

Table S4. The variants calling performances for sample HG001 based on the bechmark variants. (Note: NS, NovaSeq 6000; GL, GenoLab M; FA,FASTASeq 300; SF,SURFSeq 5000. Truth_number, The number of variants derived from the GIAB benchmark dataset. These results of variants were detected with the default parameters for all tools.)

Table S5. The performances of variant allele frequency for all datasets of sample HD832. (Note: Chr, Chromosome; POS, Position; Ref, Reference; Alt, Alternate; AF, Allele frequency provided by OncoSpan FFPE HD832; Groups R1 and R2 are biological replicates. R2.1, R2.2 and R2.3 are technical duplications; NS, NovaSeq 6000; NX, NextSeq 550; GL, GenoLab M. Gene names are listed in italics.)

Table S6. The variants of possible cause effect for all datasets across multiple platforms and softwares in sample HD832. (Note: Chr, Chromosome; POS, Position; Ref, Reference; Alt, Alternate; Average Depth(×), The average depth for all datasets; Average AF, The average Allele frequency for all datasets; Func, Functional consequences of the variant by ANNOVAR; Groups R1 and R2 are biological replicates. R2.1, R2.2 and R2.3 are technical duplications; NS, NovaSeq 6000; NX, NextSeq 550; GL, GenoLab M. The value of the each software represented the allele frequency and depth of the alternate, which separated by slash. For example, 36.11%/31, 36.11% represents the allele frequency of variant, and 31 represents the depth. Gene names are listed in italics.)
